# Skin cancer prevention behaviors, beliefs, distress, and worry among hispanics in Florida and Puerto Rico

**DOI:** 10.1186/s12889-023-17039-y

**Published:** 2023-11-13

**Authors:** John Charles A. Lacson, Brenda Soto-Torres, Steven K. Sutton, Scarlet H. Doyle, Youngchul Kim, Richard G. Roetzheim, Susan T. Vadaparampil, Peter A. Kanetsky

**Affiliations:** 1 Department of Cancer Epidemiology, H. Lee Moffitt Cancer Center & Research Institute, 12902 Magnolia Dr., MRC 213, Tampa, FL 33612 US; 2 Public Health Program, Ponce Health Sciences University, Ponce, PR USA; 3 Department of Biostatistics and Bioinformatics, H. Lee Moffitt Cancer Center and Research Institute, Tampa, FL US; 4 Department of Family Medicine, Morsani College of Medicine, University of South Florida, Tampa, FL US; 5 Department of Health Outcomes and Behavior, H. Lee Moffitt Cancer Center and Research Institute, Tampa, FL US

**Keywords:** Skin cancer, Hispanic/Latino, Prevention behaviors, Protection motivation theory, Florida, Puerto Rico

## Abstract

**Background:**

Incidence of skin cancer has been increasing among U.S. Hispanics, who often are diagnosed with larger lesions and at later stage disease. Behaviors to decrease exposure to ultraviolet radiation can reduce risk of skin cancer. We describe skin cancer prevention behaviors and psychosocial variables among Hispanic participants recruited into a skin cancer prevention trial.

**Methods:**

Self-reported Hispanic participants from eight primary care clinics in Tampa, Florida and Ponce, Puerto Rico were recruited into a randomized controlled prevention trial. Information on demographics, sun-related behaviors, and psychosocial variables were collected before intervention materials were provided. Multivariable regression models were used to compare baseline sun-related behaviors and psychosocial variables across groups defined by geographic location and language preference.

**Results:**

Participants reported low levels of intentional outdoor tanning, weekday and weekend sun exposure, and very low levels of indoor tanning. However, only a minority of participants practiced sun-protective behaviors often or always, and about 30% experienced a sunburn in the past year. Participants had low levels of recent worry and concern about skin cancer, modest levels of perceived risk and severity, and high levels of response efficacy and self-efficacy. When comparing across groups defined by geographic location and language preference, English-preferring Tampa residents (hereafter referred to as Tampeños) had the highest proportion who were sunburned (35.9%) and tended toward more risky behavior but also had higher protective behavior than did Spanish-preferring Tampeños or Puerto Ricans. Spanish-preferring Puerto Ricans had higher recent concern about skin cancer, comparative chance of getting skin cancer, and response efficacy compared to either English- or Spanish-preferring Tampeños. Spanish-preferring Tampeños had the highest levels of familism and recent distress about skin cancer.

**Conclusions:**

Our results mirror previous observations of low levels of sun-protective behavior among U.S. Hispanics compelling the need for culturally appropriate and translated awareness campaigns targeted to this population. Because Hispanics in Tampa and Puerto Rico reported modest levels of perceived risk and severity, and high levels of response efficacy and self-efficacy, interventions aiming to improve skin cancer prevention activities that are anchored in Protection Motivation Theory may be particularly effective in this population subgroup.

## Background

Incidence rates of skin cancer, including basal cell carcinoma (BCC), squamous cell carcinoma (SCC), and melanoma, have risen among U.S. Hispanics over the past several decades [1–3]. In Puerto Rico, the incidence of these three skin cancers was 106.4, 52.2, and 2.6 per 100,000 individuals, respectively, in 2005, resulting in over a 300% increase in skin cancer since 1974 [1]. Counts of skin cancer cases were determined from pathology reports obtained from 21 to 23 pathology laboratories on the island. Among U.S. Hispanics, incidence of melanoma, the most lethal skin cancer and the only skin cancer type captured in cancer registries, increased an average of 0.5% annually between 2000 and 2019, rising from 3.9 to 4.5 per 100,000 individuals [3]. These increases are further compounded by the rapid growth of the Hispanic population in the U.S. [4]. Compared to non-Hispanic Whites, Hispanics tend to be diagnosed with later stage melanoma and present with larger BCC or SCC, resulting in higher morbidity and mortality [2, 5, 6]. These disparities may be due to lack of patient and clinician awareness about skin cancer risk in Hispanics and unequal access to healthcare [7, 8].

The major environmental risk factor common to melanoma, SCC, and BCC is exposure to ultraviolet radiation [9, 10]. Thus, primary prevention strategies for these three skin cancers focus on minimizing sun exposure and intentional tanning, and wearing sunscreen and sun-protective clothing [11–13]. U.S. Hispanics and Puerto Ricans do not engage routinely in primary preventive behaviors [14–18] and have higher-than-expected frequency of experiencing sunburn in the past year despite being considered to be at low risk for sunburns [19].

One of the strongest predictors of acculturation among US Hispanics is language preference [20, 21], and previous studies have shown that skin cancer preventive behavior, beliefs, distress, and worry vary by acculturation among Hispanics. English-preferring Hispanics have lower levels of perceived risk and severity of skin cancer, and greater knowledge and less worry about skin cancer compared to Spanish-preferring Hispanics [22]. Among Puerto Ricans, those who preferred English were more likely to engage in preventive behavior against skin cancer, such as wearing sun protective clothing and wearing sunscreen, but were also more likely to have risky behaviors, such as intentional indoor or outdoor tanning, compared to those who preferred Spanish [15]. English-preferring Hispanics are also more aware of genetic testing than Spanish-preferring Hispanics [23, 24].

We conducted a prevention intervention trial among Hispanics from Tampa, Florida and Ponce, Puerto Rico to determine whether receipt of skin cancer prevention information anchored in *MC1R* genetic risk information improves skin cancer prevention activities compared to receipt of non-genetics-based prevention information. The trial was based on Protection Motivation Theory [25], which postulates that the higher the perceived risk and severity of a disease, the more likely an individual is to adopt preventive behaviors that the individual believes themselves capable to do (i.e., self-efficacy) and are effective in eliminating the threat (i.e., response efficacy).

In this report, we present baseline measures of sun-related primary prevention behaviors and skin cancer-related psychosocial variables, including those measuring Protection Motivation Theory constructs. We sought to characterize the pre-intervention levels of primary prevention behavior to better understand the potential of the population to adopt preventive behaviors recommended by the intervention. Because acculturation is associated with skin cancer knowledge, beliefs, behaviors, distress, and worry, we also assessed differences in sun-related behavior and psychosocial variables across groups defined by language preference and geographic location.

## Methods

### Participants

Full details and efficacy results of the intervention trial have been published (Lacson et al., 2022). Briefly, we recruited participants from eight primary care clinics in Tampa, FL (hereafter referred to as Tampeños), and Ponce, Juana Díaz, and Salinas, PR (Puerto Ricans) between September 2018 and January 2020. Participants were self-identified Hispanics at least 18 years old in Tampa and at least 21 years old in Puerto Rico, which are the respective ages of majority. We excluded individuals who had a skin examination within the past year, a previous diagnosis of melanoma, and more than one previous diagnosis of BCC and/or SCC.

All participants gave written informed consent. The study was conducted based on the Declaration of Helsinki and was approved by the Institutional Review Boards of the University of South Florida (Tampa, FL; Pro00020044, approved August 30, 2018), Ponce Health Sciences University (Ponce, PR; 170,807-BS, approved December 6, 2017) and the Comité de Seguimiento de la Investigación Clínica at Hospital Damas (HD 19 − 17, approved December 18, 2017).

### Study questionnaires

The baseline questionnaire (A) was completed using tablet computers while participants were in clinic, or a paper copy and prepaid envelope were given to participants who preferred to complete it at home. Baseline questionnaire A solicited information on age, sex, marital status, educational level, family history of melanoma, skin cancer, and other cancers, paid/unpaid work outdoors, health literacy [26], health numeracy [27], and untanned skin color. Baseline questionnaire A included a standardized survey that collected information on several skin cancer prevention activities [28], including: (1) time spent outside (in hours) from 10 a.m. to 4 p.m. on weekdays and weekends separately; (2) frequency of outdoor intentional tanning (never, rarely, sometimes, often, always); (3) number of sunburns; (4) number of tanning bed sessions; and (5) frequency (never, rarely, sometimes, often, always) of each of the following sun protection behaviors: (i) sunscreen usage, wearing of (ii) sunglasses, (iii) hats, and (iv) shirts that cover the shoulders; and (v) standing in the shade while outdoors or parasol usage.

Baseline questionnaire A also assessed several psychosocial variables, using questions adapted from Hay & colleagues [29]. We measured variables related to the Protection Motivation Theory: perceived risk was measured using absolute and comparative of getting skin cancer, by asking “Do you think you are likely or unlikely to get skin cancer (including melanoma, squamous cell and/or basal cell carcinoma)?” (likely, unlikely, no idea) and “Compared to the average person of your age and gender, what is the chance that you will develop melanoma, squamous cell and/or basal cell skin cancer in the future?” (well below average, below average, average, above average, well above average), respectively [30, 31]. Perceived severity was measured using the average of a 7-item 4-point Likert-type scale [30]. Response efficacy was measured using seven preventive activities (e.g., limiting sun exposure between 10am and 4pm) and asking participants how important each activity was to reduce skin cancer risk (range 1–4), while self-efficacy was measured by asking how capable they were to perform these activities (range 1–4) [29].

Other psychosocial variables included a 3-item skin cancer adaptation of Lerman’s cancer worry scale (range 1–5) [32, 33]. Recent worry about skin cancer was assessed by asking “During the past two weeks, how often have you worried about the possibility of getting skin cancer?” (rarely or never, sometimes, often, all of the time). Recent concern about skin cancer was measured by asking “During the past two weeks, how concerned have you been about the possibility of getting skin cancer?” (not at all concerned, a bit concerned, concerned, very concerned). Recent distress about skin cancer was measured using the 15-item Impact of Events scale (range 0–75) [34].

A supplemental baseline questionnaire (B) was provided for participants to complete in the clinic, at home, or online. Participants were provided with a postage prepaid return envelope. Baseline questionnaire B included questions on phenotypic traits including hair color, eye color, freckling, burnability after acute sun exposure, and ability to tan after chronic sun exposure. It also collected information on cancer fatalism, measured using the 15-item Powe Fatalism Inventory (range 0–14) [35], and familism, measured using the 18-item Attitudinal Familism Scale (range 1–4) [36].

Participants had two weeks to return both baseline questionnaires A & B. If either questionnaire was not received within one week, participants were sent an email reminder. After two weeks, a second reminder and an additional copy of the unreturned questionnaire (A and/or B) were mailed to the participant. The third and last reminder was by phone or email.

### Statistical analyses

All participants who completed baseline questionnaire were included in these analyses, regardless of whether they contributed further to the intervention study. Differences in baseline participant characteristics across groups defined by geography and language (English-preferring Tampeños, Spanish-preferring Tampeños, and Spanish-preferring Puerto Ricans) were tested using Kruskal-Wallis, ANOVA, or chi-squared tests. Participant characteristics with a statistically significant global difference (*p* ≤ 0.05) were added as covariates to multivariable models described below. Because of small numbers, the seven English-preferring Puerto Ricans recruited into the parent study were excluded from all formal analyses.

For sun-related behaviors and psychosocial variables, univariate differences across the three groups were also tested using Kruskal-Wallis, ANOVA, or chi-square tests. We examined differences in weekday and weekend sun exposure, frequency of outdoor intentional tanning, Lerman’s cancer worry scale, recent worry about skin cancer, recent concern about skin cancer, perceived risk of getting skin cancer, recent distress about skin cancer, response efficacy, self-efficacy, perceived severity, fatalism and familism. The frequencies of sun protection behaviors were examined individually as binary dependent variables (often or always vs. sometimes, rarely, or never), while absolute chance of getting skin cancer was treated as a nominal multinomial variable. Ever having a sunburn was examined as a binary dependent variable, and we assessed number of sunburns among those ever sunburned as a continuous dependent variable. We also tested the difference in the total number of sun protection behaviors practiced often or always in the past year (range 0–5).

To estimate the multivariable-adjusted population predicted marginal mean or proportion for dependent variables, and for multivariable modeling of differences across the three groups, we used linear regression for ordinal variables (weekday and weekend sun exposure, number of sunburns, frequency of outdoor intentional tanning, total number of sun protection behaviors, Lerman’s cancer worry scale, recent worry about skin cancer, recent concern about skin cancer, comparative chance of getting skin cancer, recent distress about skin cancer, response efficacy, self-efficacy, perceived severity, fatalism, familism, and sun protection behaviors; all of which were assumed to have a normal distribution), logistic regression for binary variables (frequencies of the five sun protection behaviors), and multinomial logistic regression for multinomial variables (absolute chance of getting melanoma). Pairwise comparisons were adjusted for multiple hypotheses testing using Tukey’s studentized range method. Due to the inclusion of baseline questionnaire B variables in the model, these analyses were limited to participants who answered both baseline questionnaires.

All analyses were conducted using R software (ver 4.1.0, R Foundation for Statistical Computing, Vienna, Austria, RRID:SCR_001905), RStudio (ver 1.4.1717, RStudio Team, Boston, MA, RRID:SCR_000432), and SAS (ver. 9.4., Statistical Analysis System, RRID:SCR_008567).

## Results

Of 974 participants who consented to the study, 944 (96.9%) completed baseline questionnaire A, including 497 Tampeños (121 Spanish-preferring and 376 English-preferring) and 447 Puerto Ricans (440 Spanish-preferring and 7 English-preferring). Of these, 795 (84.2%) completed supplemental questionnaire B. Overall, the mean age of participants was 45.7 years (SD = 15.7), and a majority (70.4%) were female (Table 1). Slightly over one-third (37.1%) of participants reported ever having a paid or unpaid job for a year or more in which they usually worked outdoors for at least one hour during the day.

**Table 1:** Participant characteristics by location and language preference

	Spanish-preferring		English-preferring			
	Tampa	Puerto Rico	Tampa	Puerto Rico	Overall	
Variable	(*n=*121)	(*n=*440)	(*n=*376)	(*n=*7)	(*n=*944)	***P***-value^a^
***Demographicssss***						
**Age** (Mean, SD)	38.0 (7.74)	51.4 (15.7)	41.4 (15.0)	48.1 (19.3)	45.7 (15.7)	<0.0001
**Female**	88 (72.7%)	323 (73.4%)	251 (66.8%)	3 (42.9%)	665 (70.4%)	0.03
**Marital status**						<0.0001
Single or never married	13 (10.7%)	115 (26.1%)	129 (34.3%)	1 (14.3%)	258 (27.3%)	
Married, domestic partnership, or civil union	75 (62.0%)	229 (52.0%)	194 (51.6%)	6 (85.7%)	504 (53.4%)	
Divorced, separated, or widowed	31 (25.6%)	92 (20.9%)	52 (13.8%)	0 (0%)	175 (18.5%)	
**Education**						<0.0001
Less than high school or GED	32 (26.4%)	84 (19.1%)	25 (6.6%)	0 (0%)	141 (14.9%)	
High school or GED	20 (16.5%)	50 (11.4%)	82 (21.8%)	0 (0%)	152 (16.1%)	
Some college^b^	37 (30.6%)	105 (23.9%)	103 (27.4%)	3 (42.9%)	248 (26.3%)	
Four-year college degree	14 (11.6%)	127 (28.9%)	108 (28.7%)	4 (57.1%)	253 (26.8%)	
Graduate degree or higher	9 (7.4%)	69 (15.7%)	57 (15.2%)	0 (0%)	135 (14.3%)	
**Race**						<0.0001
White	98 (81.0%)	410 (93.2%)	238 (63.3%)	7 (100%)	753 (79.8%)	
Other	23 (19.0%)	30 (6.8%)	138 (36.7%)	0 (0%)	191 (20.2%)	
**Hispanic identity**						0.0004
Central or South American, excluding Brazilian	41 (33.9%)	1 (0.2%)	67 (17.8%)	0 (0%)	109 (11.5%)	
Cuban	32 (26.4%)	1 (0.2%)	46 (12.2%)	0 (0%)	79 (8.4%)	
Dominican (Republic)	7 (5.8%)	2 (0.5%)	13 (3.5%)	0 (0%)	22 (2.3%)	
Mexican	15 (12.4%)	0 (0%)	39 (10.4%)	0 (0%)	54 (5.7%)	
Mixed	0 (0%)	3 (0.7%)	27 (7.2%)	0 (0%)	30 (3.2%)	
Other	0 (0%)	0 (0%)	20 (5.3%)	0 (0%)	20 (2.1%)	
Puerto Rican	26 (21.5%)	433 (98.4%)	164 (43.6%)	7 (100%)	630 (66.7%)	
**Family history of melanoma**	13 (10.7%)	64 (14.5%)	36 (9.6%)	1 (14.3%)	114 (12.1%)	0.08
**Family history of non-melanoma skin cancer**	6 (5.0%)	19 (4.3%)	23 (6.1%)	0 (0%)	48 (5.1%)	0.52
**Family history of other cancers**	53 (43.8%)	288 (65.5%)	165 (43.9%)	7 (100%)	513 (54.3%)	<0.0001
**Worked outdoors**	46 (38.0%)	167 (38.0%)	132 (35.1%)	5 (71.4%)	350 (37.1%)	0.58
**Health literacy**						<0.0001
Extremely confident	37 (30.6%)	85 (19.3%)	48 (12.8%)	0 (0%)	170 (18.0%)	
Quite a bit confident	43 (35.5%)	148 (33.6%)	106 (28.2%)	2 (28.6%)	299 (31.7%)	
Not at all, a little bit, or somewhat confident	38 (31.4%)	204 (46.4%)	220 (58.5%)	5 (71.4%)	467 (49.5%)	
**Health numeracy**						<0.0001
Very easy	19 (15.7%)	105 (23.9%)	122 (32.4%)	2 (28.6%)	248 (26.3%)	
Easy	73 (60.3%)	218 (49.5%)	213 (56.6%)	5 (71.4%)	509 (53.9%)	
Hard or very hard	27 (22.3%)	114 (25.9%)	39 (10.4%)	0 (0%)	180 (19.1%)	
***Pigmentation characteristics***						
**Untanned skin color**						0.06
Fair or very fair	48 (39.7%)	214 (48.6%)	137 (36.4%)	3 (42.9%)	402 (42.6%)	
Olive	8 (6.6%)	33 (7.5%)	106 (28.2%)	0 (0%)	147 (15.6%)	
Light brown	50 (41.3%)	167 (38.0%)	117 (31.1%)	4 (57.1%)	338 (35.8%)	
Dark brown or very dark	15 (12.4%)	24 (5.5%)	14 (3.7%)	0 (0%)	53 (5.6%)	
**Hair color**^c^						0.03
Red or blonde	12 (9.9%)	53 (12.0%)	33 (8.8%)	0 (0%)	98 (10.4%)	
Brown	49 (40.5%)	153 (34.8%)	195 (51.9%)	2 (28.6%)	399 (42.3%)	
Black	32 (26.4%)	132 (30.0%)	117 (31.1%)	1 (14.3%)	282 (29.9%)	
**Eye color**^c^						0.71
Blue, green, or other	12 (9.9%)	56 (12.7%)	54 (14.4%)	0 (0%)	122 (12.9%)	
Brown or black	81 (66.9%)	285 (64.8%)	289 (76.9%)	3 (42.9%)	658 (69.7%)	
**Freckling**^c^						<0.0001
None	42 (34.7%)	147 (33.4%)	210 (55.9%)	1 (14.3%)	400 (42.4%)	
Very few	37 (30.6%)	97 (22.0%)	95 (25.3%)	1 (14.3%)	230 (24.4%)	
Few to very many	16 (13.2%)	99 (22.5%)	44 (11.7%)	1 (14.3%)	160 (16.9%)	
**Burnability after acute sun exposure**^c^						0.0003
Painful to severe sunburn	37 (30.6%)	154 (35.0%)	101 (26.9%)	2 (28.6%)	294 (31.1%)	
Mild sunburn with mild tanning	32 (26.4%)	119 (27.0%)	155 (41.2%)	0 (0%)	306 (32.4%)	
No sunburn with tanning	26 (21.5%)	66 (15.0%)	91 (24.2%)	1 (14.3%)	184 (19.5%)	
**Tannability after chronic sun exposure**^c^						0.07
Very brown and deeply tanned	36 (29.8%)	167 (38.0%)	150 (39.9%)	3 (42.9%)	356 (37.7%)	
Moderate tan	35 (28.9%)	114 (25.9%)	124 (33.0%)	0 (0%)	273 (28.9%)	
Mild or no tan	23 (19.0%)	58 (13.2%)	73 (19.4%)	0 (0%)	154 (16.3%)	
***Sun-related behaviors***^*d*^						
**Weekday sun exposure** (Hrs, Mean, SD)	1.99 (1.98)	1.74 (1.65)	1.53 (1.42)	2.86 (2.04)	1.70 (1.62)	0.62
**Weekend sun exposure** (Hrs, Mean, SD)	1.89 (1.53)	1.85 (1.64)	2.26 (1.59)	2.07 (1.74)	2.02 (1.62)	<0.0001
**Ever had sunburns** (n, %)	25 (20.5%)	105 (23.9%)	147 (39.1%)	3 (42.9%)	280 (29.7%)	<0.0001
**Number of sunburns** (among those ever sunburned, Mean, SD)	1.76 (1.16)	1.71 (1.04)	1.70 (0.97)	1.67 (1.15)	1.71 (1.01)	0.98
**Outdoor intentional tanning** (Mean, SD)	1.47 (0.77)	1.48 (0.80)	1.90 (0.91)	1.43 (0.79)	1.65 (0.87)	<0.0001
**Indoor tanning**	0 (0%)	4 (0.9%)	18 (4.8%)	0 (0%)	22 (2.3%)	0.0004
**Sun protection behaviors**^e^ (Mean, SD)	1.96 (1.31)	1.80 (1.26)	2.03 (1.26)	2.43 (0.98)	1.91 (1.27)	0.03
**Wore a hat often or always**	26 (21.5%)	75 (17.0%)	59 (15.7%)	4 (57.1%)	164 (17.4%)	0.33
**Sought shade or used an umbrella often or always**	56 (46.3%)	173 (39.3%)	153 (40.7%)	3 (42.9%)	385 (40.8%)	0.39
**Wore a shirt with sleeves often or always**	70 (57.9%)	264 (60.0%)	238 (63.3%)	6 (85.7%)	578 (61.2%)	0.51
**Wore sunglasses often or always**	55 (45.5%)	193 (43.9%)	200 (53.2%)	4 (57.1%)	452 (47.9%)	0.03
**Wore sunscreen often or always**	28 (23.1%)	77 (17.5%)	108 (28.7%)	0 (0%)	213 (22.6%)	0.0009
***Psychosocial variables***						
**Skin cancer worry**	2.10 (0.87)	2.02 (0.86)	1.92 (0.76)	2.38 (1.01)	1.99 (0.83)	0.14
**Recent worry about skin cancer**						<0.0001
Rarely or never	96 (79.3%)	320 (72.7%)	320 (85.1%)	5 (71.4%)	741 (78.5%)	
Sometimes, often, or all the time	23 (19.0%)	117 (26.6%)	54 (14.4%)	2 (28.6%)	196 (20.8%)	
**Recent concern about skin cancer**						<0.0001
Not at all concerned	92 (76.0%)	288 (65.5%)	297 (79.0%)	6 (85.7%)	683 (72.4%)	
A bit concerned, concerned, or very concerned	27 (22.3%)	149 (33.9%)	76 (20.2%)	1 (14.3%)	253 (26.8%)	
**Chance of getting skin cancer (absolute)**						<0.0001
Unlikely	73 (60.3%)	166 (37.7%)	185 (49.2%)	3 (42.9%)	427 (45.2%)	
Likely	35 (28.9%)	125 (28.4%)	138 (36.7%)	2 (28.6%)	300 (31.8%)	
No idea	12 (9.9%)	144 (32.7%)	51 (13.6%)	2 (28.6%)	209 (22.1%)	
**Chance of getting skin cancer (comparative)**						<0.0001
Well below average	38 (31.4%)	70 (15.9%)	81 (21.5%)	0 (0%)	189 (20.0%)	
Below average	30 (24.8%)	102 (23.2%)	120 (31.9%)	0 (0%)	252 (26.7%)	
Average	35 (28.9%)	194 (44.1%)	147 (39.1%)	5 (71.4%)	381 (40.4%)	
Above or well above average	10 (8.3%)	70 (15.9%)	24 (6.4%)	2 (28.6%)	106 (11.2%)	
**Recent distress about skin cancer** (Mean, SD)	10.0 (13.4)	8.41 (13.2)	5.76 (9.58)	2.71 (5.47)	7.50 (12.0)	0.009
**Response efficacy** (Mean, SD)	3.33 (0.71)	3.59 (0.52)	2.99 (0.76)	3.59 (0.51)	3.32 (0.71)	<0.0001
**Self-efficacy** (Mean, SD)	3.39 (0.68)	3.44 (0.57)	3.34 (0.58)	3.76 (0.24)	3.40 (0.59)	0.006
**Perceived severity** (Mean, SD)	2.41 (0.52)	2.49 (0.41)	2.48 (0.35)	2.51 (0.45)	2.48 (0.40)	0.19
**Fatalism**^c^ (Mean, SD)	2.64 (3.10)	3.15 (2.87)	3.12 (2.83)	6.5 (3.54)	3.07 (2.89)	0.03
**Familism**^c^ (Mean, SD)	3.24 (0.36)	3.10 (0.40)	2.94 (0.39)	3.30 (0.12)	3.05 (0.41)	<0.0001

^a^*P*-values are from ANOVA, Kruskal-Wallis, chi-square, or Fisher's exact tests comparing differences across all groups after excluding Puerto Rico English-preferring participants

^b^Participants who indicated being educated outside the US were assigned the median value (some college)

^c^These variables were collected using a supplemental questionnaire that was completed by 84.2% of participants

^d^Participants were asked to report the average of these for the past 12 months

^e^Sum of all sun protection behaviors practiced often or always in the past 12 months

### Behaviors, beliefs, distress, and worry in the overall study population

Participants reported an average of 1.70 h/day (SD = 1.62) of weekday sun exposure, 2.02 h/day (SD = 1.62) of weekend sun exposure, and rare outdoor intentional tanning (M = 1.65, SD = 0.87) over the past year. Nearly a third of participants (29.7%) reported ever having a sunburn in the past 12 months, with an average of 1.71 sunburns (SD = 1.01) among those ever sunburned. Only 2.3% of participants reported indoor tanning in the past year. Wearing of sleeved shirts was practiced often or always by a majority (61.2%) of participants, nearly half (47.9%) wore sunglasses, and 40.8% sought shade or used an umbrella; less than a quarter of participants wore a hat (17.4%) or used sunscreen (22.6%) often or always. The average total number of the five sun protection behaviors practiced often or always over the past year was 1.91 (SD = 1.27).

Participants had a low average skin cancer worry (M = 1.99, SD = 0.83, Table 1). One-fifth (20.8%) of participants reported recently worrying about skin cancer sometimes to all the time, while a quarter (26.8%) reported recently being a bit to very concerned. About a third (31.8%) of participants reported being likely to get skin cancer, and 11.2% believed their chances of getting skin cancer to be above or well above the average. There was a modest level of perceived severity (M = 2.48, SD = 0.40) of skin cancer, and high levels of response efficacy (M = 3.32, SD = 0.71) and self-efficacy (M = 3.40, SD = 0.59). Participants had low mean levels of recent distress about skin cancer (M = 7.50, SD = 12.0).

### Comparison of behaviors, beliefs, distress, and worry across groups defined by geographic location and language preference

Crude differences in behaviors, beliefs, distress, and worry across Spanish-preferring Tampeños, English-preferring Tampeños, Spanish-preferring Puerto Ricans, and English-preferring Puerto Ricans are presented in Table 1.

**Fig. 1:**
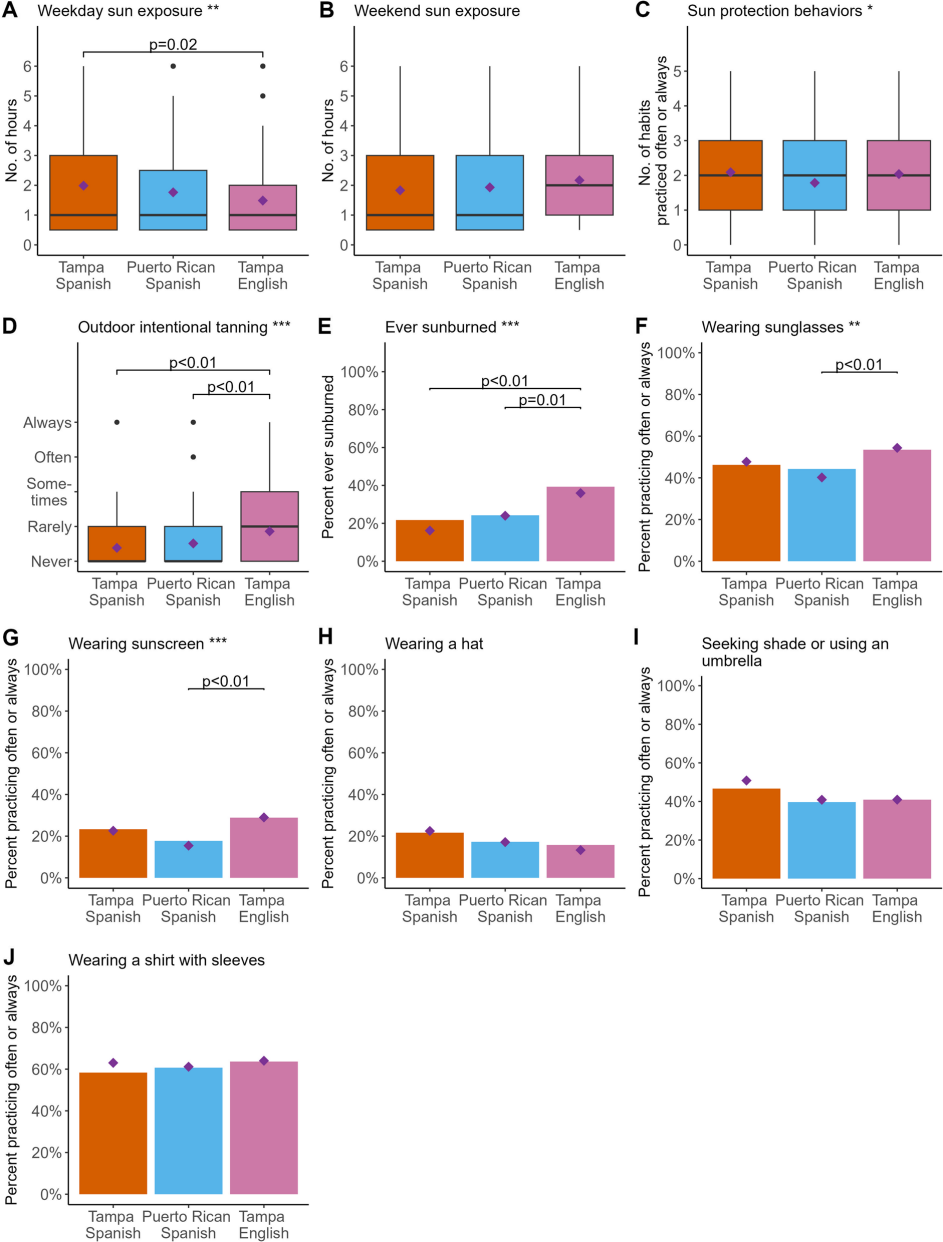
Box plots, stratified by language preference and geographic location, show the raw distribution of weekday (**A**) and weekend (**B**) sun exposure, sun protection behaviors (**C**), and outdoor intentional tanning (**D**) reported by participants over the past year. Bar plots, stratified by language preference and geographic location, show the proportion of participants reporting a sunburn (**E**), often or always wearing sunglasses (**F**), often or always wearing sunscreen (**G**), often or always wearing a hat (**H**), often or always seeking shade or using an umbrella (**I**), and often or always wearing a shirt with sleeves (**J**), over the past year. Purple diamonds represent population predicted marginal means or proportions. Asterisk(s) after the plot title provide *P*-values of the global significance from multivariate regression analyses testing differences in means/proportions across the three groups; * *p* ≤ 0.05, ** *p* ≤ 0.01, *** *p* ≤ 0.001. Brackets show statistically significant *p*-values testing pairwise differences adjusted using Tukey’s method for multiple hypotheses testing

After adjustment for age, sex, marital status, education, race, ethnic identity, family history of other cancers, health literacy, health numeracy, hair color, freckling, and burnability after sun exposure, there was a statistically significant global difference across the three groups for weekday sun exposure (*p* = 0.01, Fig. 1A), but not for weekend sun exposure (Fig. 1B). Adjusted differences in total number of sun protection behaviors (*p* = 0.04, Fig. 1C), outdoor intentional tanning (*p* < 0.0001, Fig. 1D), and ever having sunburns (*p* = 0.0002, Fig. 1E) also reached statistical significance. Among those ever sunburned, there were no significant differences in the average number of sunburns across the three groups after adjustment (*p* = 0.22). There were statistically significant adjusted global differences across groups in wearing sunglasses (*p* = 0.006, Fig. 1F) and sunscreen (*p* = 0.0009, Fig. 1G) often or always, but not for wearing a hat (Fig. 1H), seeking shade or using an umbrella (Fig. 1I), or wearing a shirt with sleeves (Fig. 1J). Pairwise comparisons indicated Spanish-preferring Tampeños had higher weekday sun exposure (M = 1.99) than English-preferring Tampeños (M = 1.49, *p* < 0.01). Both Spanish-preferring Tampeños (16.2%, *p* < 0.0001) and Spanish-preferring Puerto Ricans (24.0%, *p* < 0.0001) had a lower proportion of ever having a sunburn than English-preferring Tampeños (35.9%). Spanish-preferring Puerto Ricans (M = 1.51, *p* < 0.01) and Spanish-preferring Tampeños (M = 1.38, *p* < 0.01) also had less frequent outdoor intentional tanning than English-preferring Tampeños (M = 1.86). Spanish-preferring Puerto Ricans were less likely to wear sunglasses often or always (40.2%, *p* < 0.01) and less likely to use sunscreen (15.4%, *p* < 0.01) often or always than English-preferring Tampeños (54.4% and 29.0%, respectively). Proportions for Spanish-preferring Tampeños (47.7% and 22.6%, respectively) were intermediate to the two other groups for both behaviors.

**Fig. 2:**
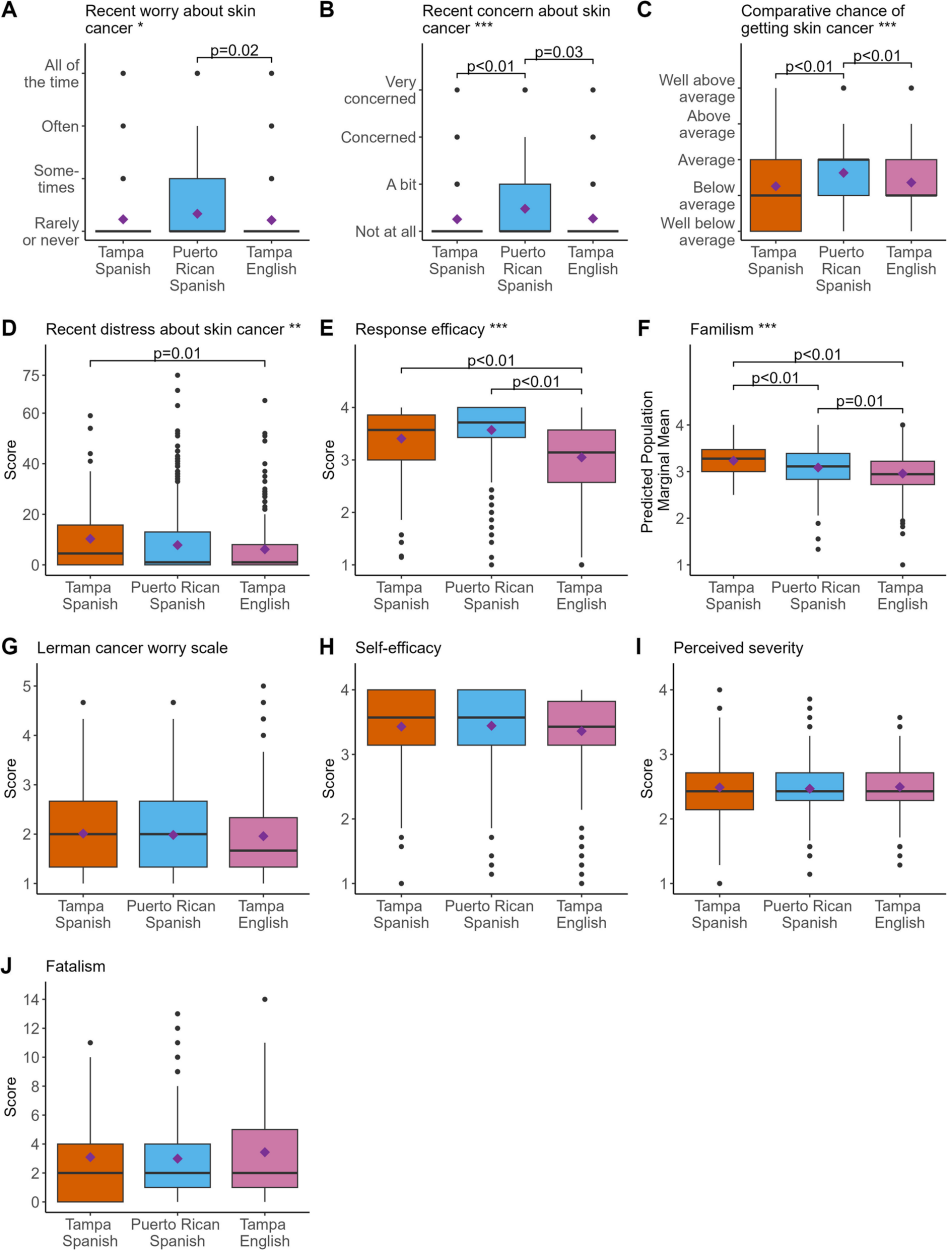
Box plots, stratified by language preference and geographic location, show the raw distribution of recent worry about skin cancer (**A**), recent concern about skin cancer (**B**), comparative chance of getting skin cancer (**C**), recent distress about skin cancer (**D**), response efficacy (**E**), familism (**F**), Lerman cancer worry scale (**G**), self-efficacy (**H**), perceived severity (**I**), and fatalism (**J**). Purple diamonds represent population predicted marginal means. Asterisk(s) after the plot title provide *P* -values of the global significance from multivariate regression analyses testing differences in means across the three groups; * *p* ≤ 0.05, ** *p* ≤ 0.01, *** *p* ≤ 0.001. Brackets show statistically significant *p*-values testing pairwise differences adjusted using Tukey’s method for multiple hypotheses testing

For psychosocial measures and after adjustment, there were statistically significant global differences across the three groups for recent worry about skin cancer (*p* = 0.047, Fig. 2A), recent concern about skin cancer (*p* = 0.0006, Fig. 2B), comparative chance of getting skin cancer (*p* = 0.001, Fig. 2C), recent distress about skin cancer (*p* = 0.01, Fig. 2D), response efficacy (*p* < 0.0001, Fig. 2E), and familism (*p* < 0.0001, Fig. 2F); but not for cancer worry (Lerman scale, Fig. 2G), self-efficacy (Fig. 2H), perceived severity (Fig. 2I), or fatalism (Fig. 2J). Pairwise comparisons showed Spanish-preferring Puerto Ricans had higher recent worry about skin cancer (M = 1.33) than English-preferring Tampeños (M = 1.21, *p* = 0.02), and they had higher recent concern about skin cancer (M = 1.48) than either Spanish-preferring (M = 1.26, *p* < 0.01) or English-preferring (M = 1.27, *p* = 0.03) Tampeños. Similarly, Spanish-preferring Puerto Ricans reported higher comparative chance of getting skin cancer (M = 2.63) than either Spanish-preferring (M = 2.26, *p* < 0.01) or English-preferring (M = 2.36, *p* < 0.01) Tampeños. Spanish-preferring Tampeños reported higher recent distress about skin cancer (M = 10.29) than English-preferring Tampeños (M = 6.14, *p* < 0.01). Spanish-preferring Puerto Ricans (M = 3.57, *p* < 0.01) and Spanish-preferring Tampeños (M = 3.41, *p* < 0.01) both had higher response efficacy than English-preferring Tampeños (M = 3.05). Spanish-preferring Tampeños reported the highest amount of familism (M = 3.23) followed by Spanish-preferring Puerto Ricans (M = 3.09) and English-preferring Tampeños (M = 2.96), and each of the three pairwise comparisons showed a statistically significantly difference (*p* ≤ 0.01).

There also was a statistically significant global difference in absolute chance of getting skin cancer across the three groups (*p* < 0.0001). Compared to participants who responded “unlikely”, both English- (OR = 0.33, 95%CI:0.26–0.41) and Spanish-preferring Tampeños (OR = 0.25, 95%CI:0.17–0.38) were statistically significantly less likely to respond with “no idea.” There were no significant differences in odds of responding with “likely” versus “unlikely.”

## Discussion

We found low to modest baseline levels of weekday and weekend sun exposure and outdoor and indoor intentional tanning among our Hispanic participants. However, about 30% of our participants reported having a sunburn in the past 12 months, with an average of almost two sunburns among those ever sunburned; and most participants in our study did not practice sun protection behaviors often or always, with an average of only two out of five sun protection behaviors being practiced often or always.

Overall, our results reflect previous literature demonstrating high rates of experiencing sunburns among Hispanics, and low prevalence of routine sun protection behaviors. Compared to a report on sunburns among Hispanics who completed the 2015 National Health Interview Survey, our study population had a similar proportion of individuals who had a sunburn in the past year [19]. Compared to reported estimates among Hispanic adults drawn from the 2010 National Health Interview Survey [17], our study population had lower routine (often or always) sunscreen use and shade-seeking. However, our population also had lower outdoor intentional tanning than a 2015 survey report on 443 Hispanic individuals [37].

English-preferring Tampeños had the lowest weekday sun exposure, and the highest frequencies of wearing sunglasses, sunscreen, and sleeved shirts. However, they also had the highest weekend sun exposure, highest frequency of outdoor intentional tanning, and the highest proportion who were ever sunburned in the past year. It is unlikely that daytime outdoor work accounts for the observed difference in lower weekday sun exposure since similar proportions of English-preferring Tampeños (35.1%), Spanish-preferring Tampeños (38%), and Puerto Ricans (38%) reported such work. These percentages are similar to the percentage of the US Hispanic workforce (35.0%) that are employed in building and grounds cleaning and maintenance, farming, construction, installation, and maintenance occupations [38], which we assume to involve substantial outdoor work. Our findings are similar to previous research that found greater knowledge and more frequent practice of sun protection behaviors, but also greater perceived suntan benefits, higher frequency of intentional tanning, and higher occurrence of sunburns among English-acculturated Hispanics compared to Spanish-acculturated Hispanics [15, 22, 39].

Hispanic participants in our study reported low skin cancer worry, recent worry, recent concern, and recent distress about skin cancer, high levels of response efficacy and self-efficacy, and a plurality reported being unlikely to get skin cancer or having below or well below average comparative risk. Yet, participants also reported modest levels of perceived severity. Two previous studies examined these psychosocial variables among U.S. Hispanics [22, 40] and Puerto Ricans [15] and reported higher perceived risk, perceived severity, and skin cancer worry; and lower fatalism about skin cancer among their participants. Because Hispanics are a heterogeneous group, and we do not necessarily expect our results to be generalizable to Hispanics outside of Florida and Puerto Rico and to Hispanic identities not represented in our study. Moreover, due to measurement differences of these constructs across studies (i.e., use of different questions and scales), a direct comparison of results is challenging.

Compared to both English- and Spanish-preferring Tampeños, Spanish-preferring Puerto Ricans had the highest recent concern about skin cancer, comparative chance of getting skin cancer, and response efficacy, and, non-significantly, the highest recent worry about skin cancer. These results are similar to previous study among U.S. Hispanics, comprised of mostly Mexicans from California and Texas, that found Spanish-acculturated Hispanics had higher perceived risk and worry compared to English-acculturated Hispanics [22]. However, recent worry, concern, and comparative chance among Spanish-preferring Tampeños were more similar to their English-preferring counterparts. Most differences in psychosocial measures between Spanish-preferring Puerto Ricans and Spanish-preferring Tampeños in our study were non-significant, except that Spanish-preferring Puerto Ricans had higher recent concern about skin cancer, comparative chance of getting skin cancer, and lower familism. These differences may have arisen due to differences in Hispanic identity between the two populations: Spanish-preferring Tampeños were a more diverse group that included significant proportions of Central or South Americans, Cubans, and Mexicans.

Because Hispanic men and individuals with low education, health literacy and health numeracy were underrepresented in our study, our findings may have limited generalizability to these groups. Similarly, our findings are likely to be most relevant to Hispanics reporting a Puerto Rican identity. It also is important to appreciate temporal trends that may have influenced reported measures obtained from participants living in Ponce, Juana Díaz, and Salinas, where Hurricane Irma and Maria struck in September 2017, which likely affected typical sun-related activities and prevention behaviors reported over the 1-year ‘think back’ timeframe anchoring our baseline questions.

## Conclusions

Although Hispanics in Florida and Puerto Rico have low levels of risky sun-related behavior, they also have suboptimal levels of sun-protective behavior and had a high occurrence of sunburn in the past year. Thus, we recommend culturally appropriate Spanish-language awareness campaigns about skin cancer risk targeted at this population. Because sun-protective behaviors were particularly low among English-preferring Tampeños, educational materials in both Spanish language and English language may optimize uptake of prevention messages. Hispanics in Florida and Puerto Rico also reported modest levels of perceived risk and severity, and high levels of response efficacy and self-efficacy, making them an ideal target for an intervention anchored in Protection Motivation Theory. We also found other differences in sun-related behavior and psychosocial variables by location and language preference that may inform future interventions and prevention initiatives.

## Data Availability

Data used in this manuscript can be obtained upon request from the corresponding author.

## Abbreviations

BCC: Basal cell carcinoma
FL: Florida
M: Mean
PR: Puerto Rico
SCC: Squamous cell carcinoma
SD: Standard deviation
